# Supplementary material for: Lens transmittance shapes ultraviolet sensitivity in the eyes of frogs from diverse ecological and phylogenetic backgrounds
Source: Proc Biol Sci. 2020 Jan 8;287(1918):20192253. doi: 10.1098/rspb.2019.2253 (PMC7003468; doi:10.1098/rspb.2019.2253)
Supplement: Supplementary methods and results [file rspb20192253supp1.pdf]

SUPPLEMENTARY MATERIAL 1

Yovanovich CAM, Pierotti MER, Kelber A, Jorgewich-Cohen G, Ibáñez R & Grant T.

Lens transmittance shapes UV sensitivity in the eyes of frogs from diverse ecological and phylogenetic backgrounds

Contents

A. Specimens used for lens and cornea transmittance measurements 1

B. Eye size data 2

C. Comparison of different eye size measuring methods 3

D. Pupil shape scoring 4

E. Species replacement in the phylogenetic tree 4

F. Phylogenetic tree used for statistical analyses 4

G. Corneal transmittance 5

H. Lens transmittance curves of species not shown in Figure 3 of the main text 6

I. Statistical analyses 7

J. Lens transmittance vs. eye size in a subset of anuran species with putatively unpigmented lenses 8

REFERENCES 8

A. Specimens used for lens and cornea transmittance measurements

Species	Sex	Source	Locality (State)	Country
Bombina orientalis	NR	CB	Lund	Sweden
Brachycephalus ephippium	F	FT	Mogi das Cruzes (SP)	Brazil
Brachycephalus ephippium	M	FT	Mogi das Cruzes (SP)	Brazil
Ischnocnema parva	M	FT	Boracéia (SP)	Brazil
Craugastor fitzingeri	M	FT	Gamboa	Panama
Thoropa taophora	F	FT	São Sebastião, (SP)	Brazil
Thoropa taophora	M	FT	São Sebastião (SP)	Brazil
Hylodes asper	M	FT	São Sebastião (SP)	Brazil
Hylodes phyllodes	M	FT	Boracéia (SP)	Brazil
Hylodes pipilans	M	FT	São Sebastião (SP)	Brazil
Agalychnis callidryas	M	FT	Gamboa	Panama
Pithecopus hypochondrialis	M	FT	Fazenda Treviso (PA)	Brazil
Bokermannohyla astartea	M	FT	Boracéia (SP)	Brazil
Bokermannohyla astartea	M	FT	Boracéia (SP)	Brazil
Bokermannohyla hylax	M	FT	Boracéia (SP)	Brazil
Boana geographica	F	FT	Fazenda Treviso (PA)	Brazil
Boana geographica	F	FT	Fazenda Treviso (PA)	Brazil
Boana rosenbergi	M	FT	Gamboa	Panama
Boana rosenbergi	M	FT	Gamboa	Panama
Scinax ruber	M	FT	Gamboa	Panama
Scinax ruber	M	FT	Gamboa	Panama
Xenohyla truncata	NR	FT	Maricá (RJ)	Brazil

Species	Sex	Source	Locality (State)	Country
Dendropsophus ebraccatus	M	FT	Gamboa	Panama
Dendropsophus ebraccatus	M	FT	Gamboa	Panama
Dendropsophus microcephalus	M	FT	Gamboa	Panama
Dendropsophus microcephalus	M	FT	Gamboa	Panama
Engystomops pustulosus	M	FT	Gamboa	Panama
Engystomops pustulosus	M	FT	Gamboa	Panama
Physalaemus cuvieri	NR	FT	Alto Paraiso de Goiás (GO)	Brazil
Physalaemus cuvieri	NR	FT	Alto Paraiso de Goiás (GO)	Brazil
Physalaemus cuvieri	NR	FT	Alto Paraiso de Goiás (GO)	Brazil
Leptodactylus insularum	M	FT	Gamboa	Panama
Leptodactylus insularum	M	FT	Gamboa	Panama
Leptodactylus fragilis	F	FT	Gamboa	Panama
Leptodactylus fragilis	F	FT	Gamboa	Panama
Leptodactylus tapiti	M	FT	Alto Paraiso de Goiás (GO)	Brazil
Leptodactylus tapiti	M	FT	Alto Paraiso de Goiás (GO)	Brazil
Teratohyla spinosa	M	FT	Soberania National Park	Panama
Teratohyla spinosa	NR	FT	Soberania National Park	Panama
Cochranella granulosa	M	FT	Soberania National Park	Panama
Cochranella granulosa	M	FT	Soberania National Park	Panama
Allobates femoralis	M	FT	Fazenda Treviso (PA)	Brazil
Adelphobates castaneoticus	M	FT	Fazenda Treviso (PA)	Brazil
Dendrobates auratus	M	FT	Ancon	Panama
Oophaga pumilio	F	FT	Bocas del Toro	Panama
Atelopus varius	F	CB	Gamboa Amphibian Recovery Center	Panama
Atelopus varius	F	CB	Gamboa Amphibian Recovery Center	Panama
Rhinella icterica	F	FT	São Paulo (SP)	Brazil
Elachistocleis panamensis	NR	FT	Gamboa	Panama

Abbreviations: M=Male; F=Female; NR=Not recorded; CB=Captive breeding; FT=Field trip.

B. Eye size data

Species	Eye length (mm)	Source
Bombina orientalis	4.1	MZUSP 115014, 59118, 59119
Brachycephalus ephippium	1.7	Thais Condez*
Ischnocnema parva	2.0	Mariane Targino*
Craugastor fitzingeri	5.8	[1]
Thoropa taophora	6.3	Ariadne F. Sabbag*
Hylodes asper	5.4	[2]
Hylodes phyllodes	3.8	[2]
Hylodes pipilans	3.1	[4]
Agalychnis callidryas	5.1	[6]
Pithecopus hypochondrialis	4.5	[7]
Bokermannohyla astartea	4.3	[9]
Bokermannohyla hylax	6.4	MZUSP 71293, 153653, 153655, 98115
Boana geographica	4.4	[12]
Boana rosenbergi	7.6	[13,14]
Scinax ruber	3.2	[16]

Species	Eye length (mm)	Source
Xenohyla truncata	3	[18]
Dendropsophus ebraccatus	3.1	MZUSP 101425
Dendropsophus microcephalus	2.7	MZUSP 126217, 126218, 92538
Engystomops pustulosus	2.6	MZUSP 135118, 135119, 5606
Physalaemus cuvieri	2.9	MZUSP 151705, 151698, 151713, 151717, 151704, 151693, 151680, 151696, 151701, 151697, 151688, 151679, 151689
Leptodactylus insularum	3.5	MZUSP 5454, 143068
Leptodactylus fragilis	3	MZUSP 143063, 143064
Leptodactylus tapiti	2.3	MZUSP 74314, 74315, 74325, 74326, 74327, 74328, 74329, 74332
Teratohyla spinosa	3.2	Marco A. Rada*
Cochranella granulosa	2.8	Roberto Ibáñez
Allobates femoralis	3.2	[3]
Aldephobates castaneoticus	2.6	[5]
Dendrobates auratus	2.8	MZUSP 100169, 100170, 100171, 100172, 100173, 100174, 100175, 100176
Oophaga pumilio	2.2	[8]
Atelopus varius	3.3	[10]
Rhinella ornata	6.4	[11]
Rhinella icterica	11.5	MZUSP 123383, 123386, 123536, 123543
Bufo bufo	5	[15]
Elachistocleis panamensis	1.7	[17]
Lithobates catesbeianus	8.2	[19]
Lithobates pipiens	6.3	[20]
Rana temporaria	4.4	[21]

(*) Unpublished data provided by the authors. MZUSP: Zoology Museum of University of São Paulo (Brazil). The numbers indicate the specimen IDs in the Herpetology collection.

C. Comparison of different eye size measuring methods

To validate the use of eye sizes available in the taxonomic literature (i.e. measured as the distance between anterior and posterior edge of the eye in intact specimens), we compared the values that we collected with those reported for the axial diameter of enucleated eyes in the species for which they were available [22]. The linear regression shows an excellent fit, demonstrating that eye sizes as reported in species' descriptions can be used as a valid proxy for actual axial lengths.

Species	Eye size, intact specimens (mm)	Axial length, enucleated eye (mm)
Bombina orientalis	4.1	4.3
Brachycephalus ephippium	1.7	2.2
Boana rosenbergi	7.6	8
Atelopus varius	3.3	3.3
Dendrobates auratus	2.8	3.1
Oophaga pumilio	2.2	2.6
Teratohyla spinosa	2.8	3.0
Engystomops pustulosus	2.6	3.1
Physalaemus cuvieri	2.9	3.2
Elachistocleis panamensis	1.7	1.8

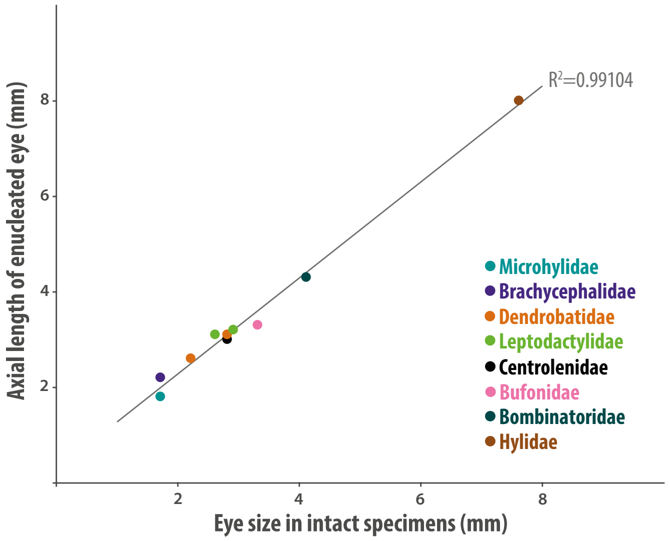

D. Pupil shape scoring

For pupil shape, we visually inspected photographs of each of the species available online and scored each of them on a dichotomic scale. The two scores were defined as "round" for those pupils that are either completely circular or just slightly oval/elliptical (and thus cover the periphery of the lens when contracting), and "elongate" for those that are clearly oval or slit-shaped (and thus contract proportionately more along one axis, while in the perpendicular axis most of the lens remains exposed to light). The threshold for "slightly oval" vs. "clearly oval" was set at:

$$\frac{Vertical\ pupil\ length}{Horizontal\ pupil\ length} = 0.75$$

E. Species replacement in the phylogenetic tree

Seven of the species in our dataset were absent in the tree from Pyron [23], thus we accommodated them as follows:

(1) We replaced *Rhinella crucifer* with *R. ornata*, which was a synonym of *R. crucifer* until it was resurrected by Baldissera and coworkers [11], and the two species are phylogenetically proximate in the *R. crucifer* species group [24].

(2) We replaced *Leptodactylus longirostris* with *L. fragilis*, both of which are in the same clade of three species in the *L. fuscus* species group [25].

(3) We replaced *L. bolivianus* with its sister species in the *L. latrans* species group, *L. insularum* [25].

(4) We replaced *L. gracilis* with *L. tapiti*; among the species in Pyron's tree [23], *L. tapiti* is phylogenetically closest to *L. gracilis* in the *L. fuscus* species group [25].

(5) We replaced *Hylodes dactylocinus* with the closely related species *H. asper* ([26]; R. Montesinos and T. Grant, unpublished data).

(6) Given their resemblance and close relationship ([4]; R. Montesinos and TG, unpublished data), we inserted *H. pipilans* as sister to *H. phyllodes* with branch lengths of zero and used the original *H. phyllodes* branch length as the branch length for the inclusive clade.

(7) Finally, we replaced *Elachistocleis bicolor* with *E. panamensis*, which is the sister species of the *E. bicolor* + *E. ovalis* clade [27].

F. Phylogenetic tree used for statistical analyses

Modified from [23] to include only the species included in the present study (see main text and S1E, above).

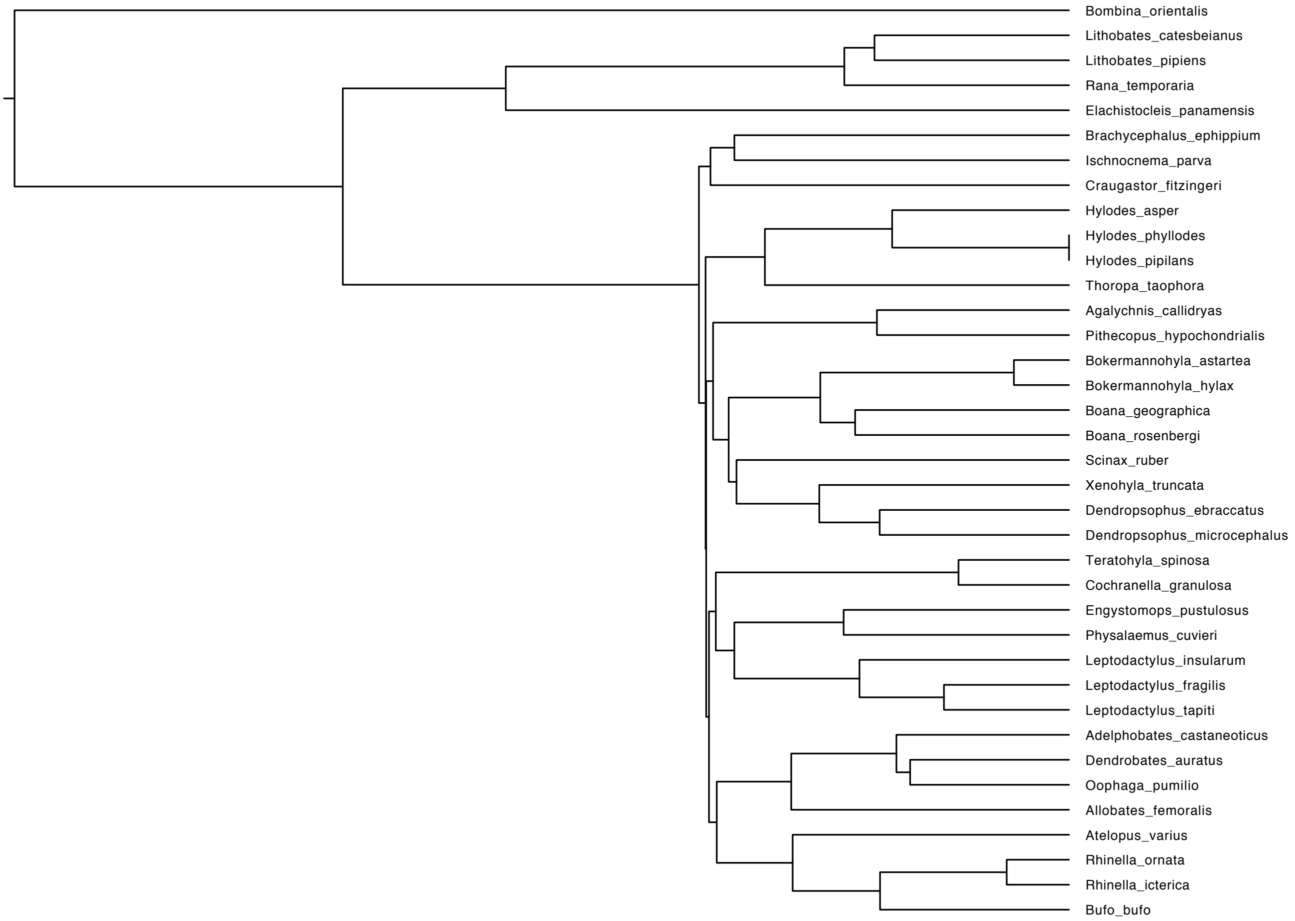

G. Corneal transmittance

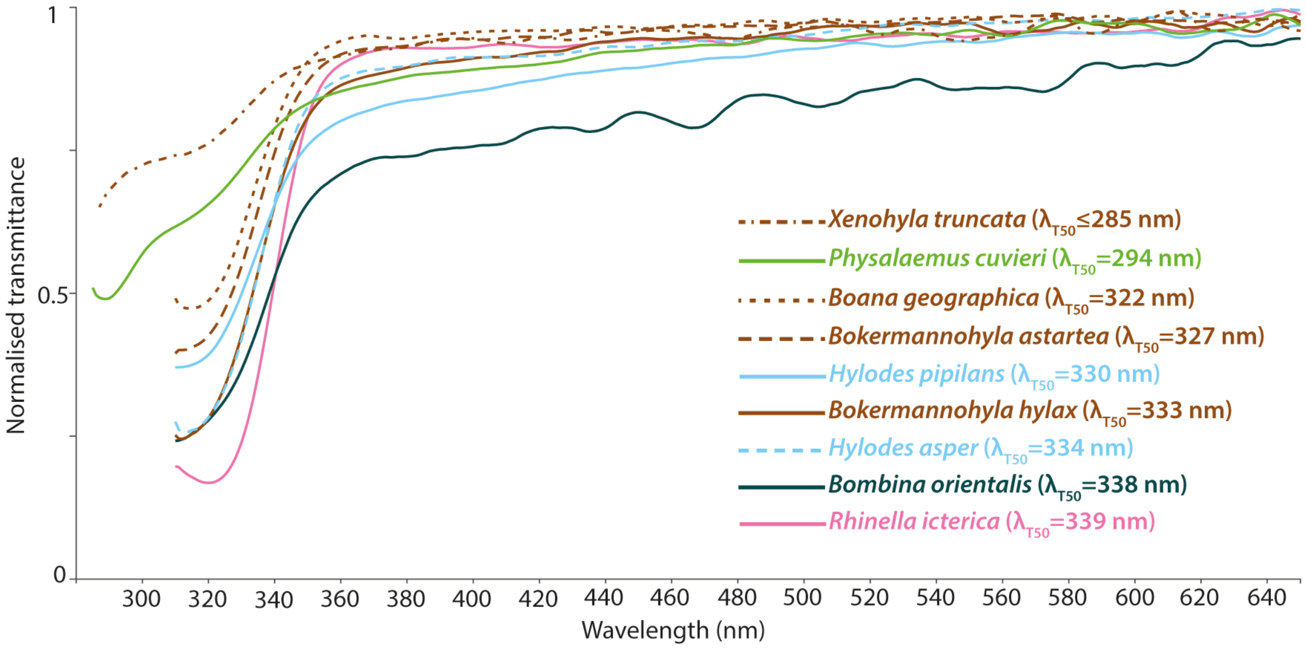

H. Lens transmittance curves of species not shown in Figure 3 of the main text

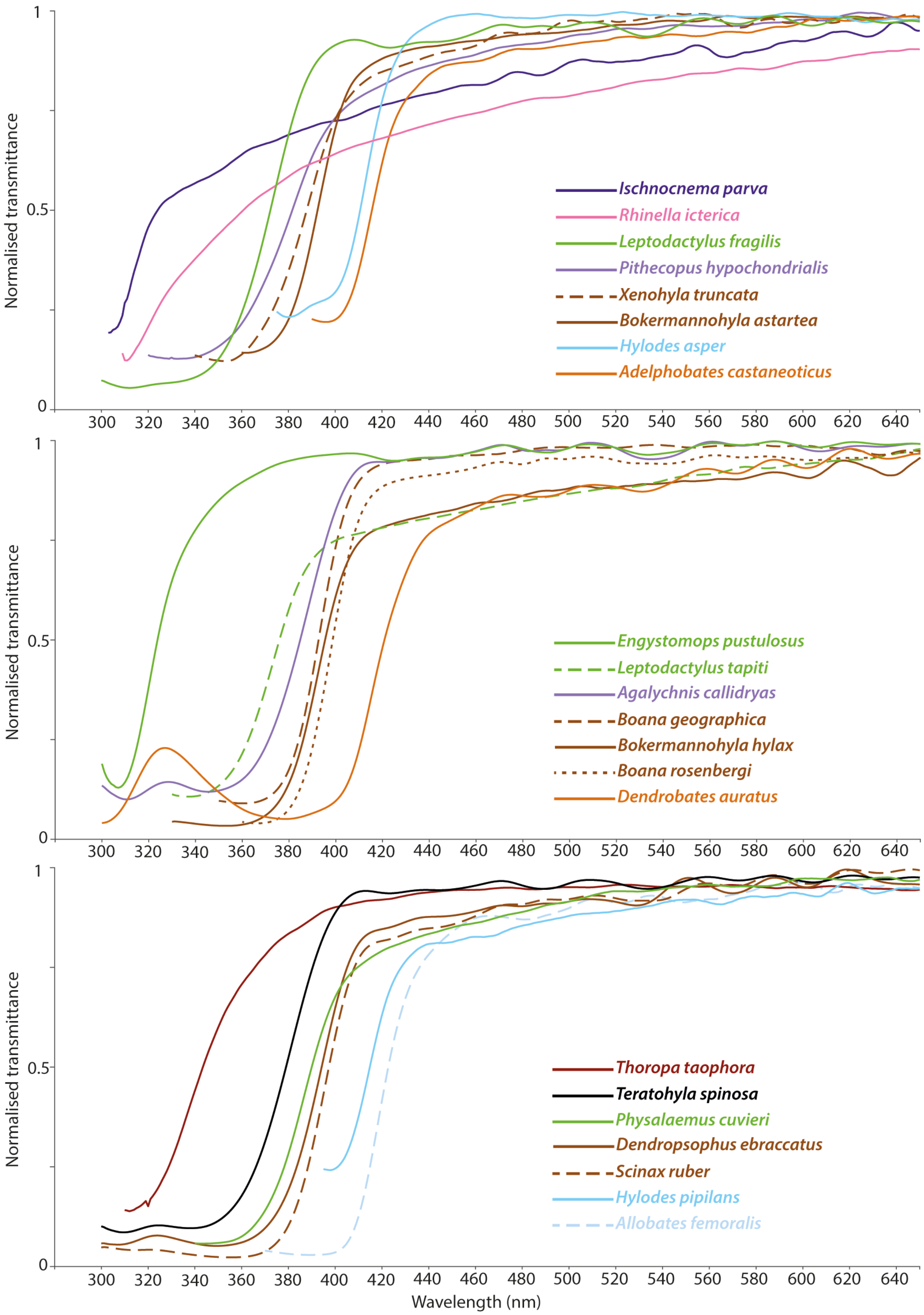

I. Statistical analyses

Results (regression coefficients, b, and corresponding p-values) of phylogenetic generalized linear mixed model analysis of binary data using the model Lens λ_{T50} ~ Eye size, excluding *Bombina orientalis*.

Variable	b	p
Eye size (λ = 0)	0.416	0.8664
Eye size (λ = 1)	0.0137215	0.2498

Results (regression coefficients, b, and corresponding p-values) of phylogenetic generalized linear mixed model analysis of binary data using the model Diel pattern ~ Lens λ_{T50} + Eye size + Pupil shape, including *Bombina orientalis*.

Variable	b	p
Lens λ_{T50}	0.031269	0.1640
Eye size	-0.568173	0.3113
Pupil shape	-1.219676	0.4019

Results (regression coefficients, b, and corresponding p-values) of phylogenetic generalized least squares analysis of continuous data using the model Elevation ~ Lens λ_{T50} + Eye size + Pupil shape, including *Bombina orientalis*.

Variable	Pagel's λ = 0		Pagel's λ = 0.1		Pagel's λ = 0.5		Pagel's λ = 1	
	b	p	b	p	b	p	b	p
Lens λ_{T50}	-0.4589	0.8399	-0.4646	0.8383	-0.45261	0.8624	1.43842	0.6183
Eye size	26.0041	0.5208	26.0506	0.5204	25.58508	0.5454	5.38262	0.9089
Pupil shape	-22.9854	0.9012	-23.2553	0.9001	-43.06161	0.8249	-70.46774	0.7386

Results (regression coefficients, b, and corresponding p-values) of phylogenetic generalized least squares analysis of continuous data using the model Latitude ~ Lens λ_{T50} + Eye size + Pupil shape, including *Bombina orientalis*.

Variable	Pagel's λ = 0		Pagel's λ = 0.1		Pagel's λ = 0.5		Pagel's λ = 1	
	b	p	b	p	b	p	b	p
Lens λ_{T50}	-0.10722	0.1223	-0.10626	0.1253	-0.07397	0.2913	0.071184	0.2670
Eye size	1.60505	0.1909	1.58655	0.1949	0.87073	0.4402	-1.752291	0.0981
Pupil shape	3.97034	0.4766	3.95965	0.4765	3.20465	0.5371	3.063991	0.5116

Results (regression coefficients, b, and corresponding p-values) of phylogenetic generalized linear mixed model analysis of binary data using the model Diel pattern ~ Lens λ_{T50} + Eye size + Pupil shape, excluding *Bombina orientalis*.

Variable	b	p
Lens λ_{T50}	0.031062	0.1710
Eye size	-0.567791	0.3124
Pupil shape	-1.219609	0.4031

Results (regression coefficients, b, and corresponding p-values) of phylogenetic generalized least squares analysis of continuous data using the model Elevation ~ Lens λ_{T50} + Eye size + Pupil shape, excluding *Bombina orientalis*.

Variable	Pagel's λ = 0		Pagel's λ = 0.1		Pagel's λ = 0.5		Pagel's λ = 1	
	b	p	b	p	b	p	b	p
Lens λ_{T50}	-1.3779	0.5995	-1.3698	0.6023	-0.7672	0.7950	1.8885	0.5730
Eye size	25.3422	0.5346	25.4029	0.5342	24.9619	0.5646	9.6770	0.8420
Pupil shape	-12.3170	0.9475	12.8484	0.9453	-43.7151	0.8279	-13.3644	0.9520

Results (regression coefficients, b, and corresponding p-values) of phylogenetic generalized least squares analysis of continuous data using the model Latitude ~ Lens λ_{T50} + Eye size + Pupil shape, excluding *Bombina orientalis*.

Variable	Pagel's λ = 0		Pagel's λ = 0.1		Pagel's λ = 0.5		Pagel's λ = 1	
	b	p	b	p	b	p	b	p
Lens λ_{T50}	-0.023462	0.7481	-0.023541	0.7469	-0.022762	0.7583	0.0766823	0.2623
Eye size	1.665369	0.1488	1.639267	0.1542	0.741399	0.4951	-1.6569201	0.0992
Pupil shape	2.998050	0.5667	2.994924	0.5661	2.345081	0.6419	1.5587347	0.7291

J. Lens transmittance vs. eye size in a subset of anuran species with putatively unpigmented lenses

Each data point represents one species, and the data point with a black outline was obtained from a previous study [28].

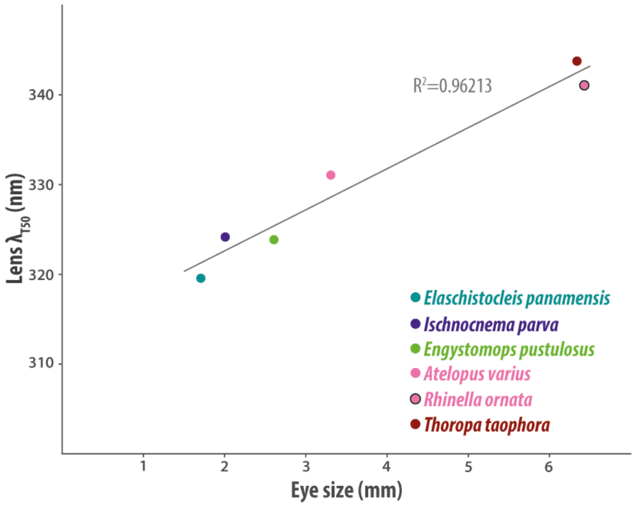

REFERENCES

1. Savage JM. 1974 On the Leptodactylid Frog Called *Eleutherodactylus palmatus* (Boulenger) and the Status of *Hylodes fitzingeri* O. Schmidt. *Herpetologica* **30**, 289–299.
2. Canedo C. 2008 Revisão Taxonômica de *Hylodes* Fitzinger, 1826 (Anura, Hylodidae). Unpublished PhD Thesis, Universidade Federal do Rio de Janeiro, Rio de Janeiro, Brazil.
3. Simões PI. 2010 Diversificação do complexo *Allobates femoralis* (Anura, Dendrobatidae) em florestas da Amazônia brasileira: desvendando padrões atuais e históricos. PhD, Instituto Nacional de Pesquisa da Amazonia, Manaus, Brasil. See https://bdtd.inpa.gov.br/handle/tede/1881.
4. Canedo C, Pombal JP. 2007 Two new species of torrent frog of the genus *Hylodes* (Anura, Hylodidae) with nuptial thumb tubercles. *Herpetologica* **63**, 224–235. (doi:10.1655/0018-0831(2007)63[224:TNSOTF]2.0.CO;2)
5. Caldwell JP, Myers CA. 1990 A new poison frog from Amazonian Brazil: with further revision of the quinquevittatus group of Dendrobates. *Am. Mus. Novit.* **2988**, 1–21.
6. Savage JM, Heyer WR. 1967 Variation and distribution in the tree-frog genus *Phyllomedusa* in Costa Rica, central America. *Beitrage Zur Neotropischen Fauna* **5**, 111–131. (doi:10.1080/01650526709360400)

7. Haga IA, Andrade FS de, Bruschi DP, Recco-Pimentel SM, Giaretta AA. 2017 Unrevealing the leaf frogs Cerrado diversity: A new species of *Pithecopus* (Anura, Arboranae, Phyllomedusidae) from the Mato Grosso state, Brazil. *PLOS ONE* **12**, e0184631. (doi:10.1371/journal.pone.0184631)
8. Batista A, Köhler G. 2008 Variation in *Oophaga pumilio* (Amphibia: Anura: Dendrobatidae) in western Panama. *Salamandra* **44**, 225–234.
9. Bokermann WCA. 1967 *Hyla astartea*, nova especie da Serra do Mar em Sao Paulo (Amphibia, Hylidae). *Rev. Bras. Biol.* **27**, 157–158.
10. Lötters S, Böhme W, Günther R. 1998 Notes on the Type Material of the Neotropical Harlequin Frogs *Atelopus varius* (Lichtenstein & Martens, 1856) and *Atelopus cruciger* (Lichtenstein & Martens, 1856) Deposited in the Museum für Naturkunde of Berlin (Anura, Bufonidae). *Zoosystematics Evol.* **74**, 173–184. (doi:10.1002/mmnz.19980740203)
11. Baldissera FA, Caramaschi U, Haddad CFB. 2004 Review of the *Bufo crucifer* species group, with descriptions of two new related species (Amphibia, Anura, Bufonidae). *Arq. Mus. Nac.* **62**, 255–282.
12. Fouquet A *et al.* 2016 Cryptic diversity in the *Hypsiboas semilineatus* species group (Amphibia, Anura) with the description of a new species from the eastern Guiana Shield. *Zootaxa* **4084**, 79–104. (doi:10.11646/zootaxa.4084.1.3)
13. Thornton WA. 1964 The Frog *Hyla rosenbergi* in Colombia, South America. *Herpetologica* **20**, 188–191.
14. Duellman WE. 1970 *The hylid frogs of Middle America*. Museum of Natural History, University of Kansas.
15. Fyhrquist N, Govardovskii V, Leibrock C, Reuter T. 1998 Rod pigment and rod noise in the European toad *Bufo bufo*. *Vision Res.* **38**, 483–486. (doi:10.1016/S0042-6989(97)00177-6)
16. Duellman WE, Wiens JJ. 1993 Hylid frogs of the genus *Scinax* Wagler, 1830, in Amazonian Ecuador and Peru. *Occas. Pap. Mus. Nat. Hist. Univ. Kans.* **153**, 1–57.
17. Dunn ER, Trapido H, Evans H. 1948 A new species of the microhylid frog genus *Chiasmocleis* from Panama. *Am. Mus. Novit.* **1376**, 1–8.
18. Izecksohn E. 1959 Uma nova espécie de '"Hylidae"'da baixada fluminense, Estado de Rio de Janeiro, Brasil (Amphibia, Anura). *Rev. Bras. Biol.* **19**, 259–263.
19. Spear PA, Boily M, Giroux I, DeBlois C, Leclair MH, Levasseur M, Leclair R. 2009 Study design, water quality, morphometrics and age of the bullfrog, *Rana catesbeiana*, in sub-watersheds of the Yamaska River drainage basin, Québec, Canada. *Aquat. Toxicol.* **91**, 110–117. (doi:10.1016/j.aquatox.2008.09.011)
20. Feinberg JA, Newman CE, Watkins-Colwell GJ, Schlesinger MD, Zarate B, Curry BR, Shaffer HB, Burger J. 2014 Cryptic Diversity in Metropolis: Confirmation of a New Leopard Frog Species (Anura: Ranidae) from New York City and Surrounding Atlantic Coast Regions. *PLOS ONE* **9**, e108213. (doi:10.1371/journal.pone.0108213)
21. Speybroeck J, Beukema W, Bok B, Voort JVD. 2016 *Field Guide to the Amphibians and Reptiles of Britain and Europe*. Bloomsbury Publishing.
22. Ritland SM. 1982 The allometry of the vertebrate eye. PhD, University of Chicago, Illinois, USA.
23. Pyron RA. 2014 Biogeographic Analysis Reveals Ancient Continental Vicariance and Recent Oceanic Dispersal in Amphibians. *Syst. Biol.* **63**, 779–797. (doi:10.1093/sysbio/syu042)
24. Thomé MTC, Zamudio KR, Giovanelli JGR, Haddad CFB, Baldissera FA, Alexandrino J. 2010 Phylogeography of endemic toads and post-Pliocene persistence of the Brazilian Atlantic Forest. *Mol. Phylogenet. Evol.* **55**, 1018–1031. (doi:10.1016/j.ympev.2010.02.003)
25. de Sá RO, Grant T, Camargo A, Heyer WR, Ponssa ML, Stanley E. 2014 Systematics of the Neotropical Genus *Leptodactylus* Fitzinger, 1826 (Anura: Leptodactylidae): Phylogeny, the Relevance of Non-molecular Evidence, and Species Accounts. *South Am. J. Herpetol.* **9**. (doi:10.2994/SAJH-D-13-00022.1)
26. Pavan D, Narvaes P, Rodrigues MT. 2001 A new species of leptodactylid frog from the Atlantic Forests of southeastern Brazil with notes on the status and on the speciation of the *Hylodes* species groups. *Papéis Avulsos Zool.* **41**, 407–425.
27. de Sá RO, Streicher JW, Sekonyela R, Forlani MC, Loader SP, Greenbaum E, Richards S, Haddad CFB. 2012 Molecular phylogeny of microhylid frogs (Anura: Microhylidae) with emphasis on relationships among New World genera. *BMC Evol. Biol.* **12**, 241. (doi:10.1186/1471-2148-12-241)
28. Yovanovich CAM, Grant T, Kelber A. 2019 Differences in ocular media transmittance in classical frog and toad model species and its impact on visual sensitivity. *J. Exp. Biol.* **222**, jeb204271. (doi:10.1242/jeb.204271)
